# Physiologically Based Pharmacokinetics Model in Pregnancy: A Regulatory Perspective on Model Evaluation

**DOI:** 10.3389/fped.2021.687978

**Published:** 2021-06-23

**Authors:** Paola Coppola, Essam Kerwash, Susan Cole

**Affiliations:** Medicines and Healthcare Products Regulatory Agency, London, United Kingdom

**Keywords:** physiologically-based pharmacokinetics modelling, pharmacokinetics, pregnancy PBPK, foetal PBPK, breastfeeding PBPK, PBPK qualification, regulatory submissions

## Abstract

Physiologically based pharmacokinetics (PBPK) modelling is widely used in medicine development and regulatory submissions. The lack of clinical pharmacokinetic data in pregnancy is widely acknowledged; therefore, one area of current interest is in the use of PBPK modelling to describe the potential impact of anatomical and physiological changes during pregnancy on the medicine's pharmacokinetics. PBPK modelling could possibly represent a predictive tool to support the medicine benefit–risk decision and inform dose adjustment in this population and also to investigate medicine levels in the foetus to support the risk assessment to the foetus. In the context of regulatory application, there are, however, a number of considerations around model evaluation, and this should be tailored to the model purpose, in order to inform the confidence in the model for the intended application. A number of gestational age-related physiological changes are expected to alter the pharmacokinetics of medicines during pregnancy, and there are uncertainties on some parameters; therefore, well-qualified models are needed to improve assurance in the model prediction before this approach can be used to inform with confidence high-impact decisions as part of regulatory submissions.

## Introduction

Although drug labels generally recommend to avoid use of medicines in pregnancy and breastfeeding, pharmacological treatment may be necessary for some medical conditions. The lack of clinical pharmacokinetics (PK) data in pregnancy is widely acknowledged; therefore, one area of current interest is in the use of PBPK modelling to describe the potential impact of anatomical and physiological changes during pregnancy on the medicine's PK. PBPK modelling could represent a predictive tool to support the medicine benefit–risk decision and inform dose adjustment in this population. PBPK may also be useful to investigate medicine levels in the foetus to support the risk assessment to the foetus and in breast milk to inform exposure to infants.

Using a mathematical approach, PBPK models predict the expected medicine levels in the target population, as well as how physiological changes may alter those levels. There are, however, a number of considerations around the modelling before it can be used to inform clinical practice or regulatory submissions with confidence. This article provides an overview of considerations around the potential use of modelling to inform clinical practice with confidence, and considerations are provided regarding the qualification process usually required to support high-impact regulatory uses.

## Pregnancy Physiologically Based Pharmacokinetics Models

The physiological changes occurring in women during pregnancy may alter the absorption, distribution, metabolism and excretion (ADME) of medicines. Oral drug absorption may be delayed during pregnancy, and decreased levels of plasma proteins albumin and α1-acid glycoprotein may lead to decreased drug plasma protein binding and increased levels of unbound drugs. Moreover, protein binding of drugs, hepatic blood flow and hepatic enzyme activity are altered during pregnancy; and this may affect the elimination pathway of hepatically cleared drugs (1). As a consequence of altered metabolic enzymes activity, the blood concentrations of drugs metabolised through CYP2D6, CYP3A4, CYP2A6, UGT1A4, and UGT2B7 are expected to decrease during pregnancy as compared with those in non-pregnant subjects, while concentrations of drugs that are substrates of other enzymes, e.g., CYP1A2 and CYP2C19, are expected to increase in pregnant women (2).

PBPK modelling can be used as a predictive tool to provide an understanding of drugs disposition in pregnant women. Most gestational age-dependent physiological changes, including the development of the foetal placental compartment, may be incorporated in the model to allow an understanding of the impact of those changes on the PK of medicines in pregnant women compared with non-pregnant subject. Simcyp™ Simulator (Simcyp Ltd, Sheffield, UK, http://www.simcyp.com), GastroPlus™ (Simulations Plus Inc., Lancaster, CA, USA) and PK-Sim® (http://www.open-systems-pharmacology.org/) are PBPK platforms; and all have pregnancy models to predict exposure in pregnancy populations at different stages of pregnancy based on the physiological changes that occur.

The European Medicines Agency (EMA) and US Food and Drug Administration (FDA) recommend that, where possible, PK and pharmacodynamic (PD) studies should be conducted in pregnant women to understand how pregnancy affects the blood levels of medicines commonly used and to inform dosing regimen of medicines to be used in pregnancy (3, 4). The importance of PK studies including pregnant patients has been highlighted in the FDA's draft guidance (4), where the use of PBPK is suggested to support clinical study design in this vulnerable population.

PBPK models have been seen in regulatory submissions by regulators in Europe and the USA; and in some cases, models have been accepted to replace clinical studies and to inform the SmPC (5–7). However, pregnancy PBPK models have, to date, been seen in a very limited number of submissions in Europe.

PBPK may support the understanding of drug systemic exposures in pregnant population and be used to optimise the design of PK clinical trials for investigational medicines in this population. Given the sparsity of data and the need to, on occasions, dose pregnant women, the potential of PBPK modelling to inform this dosing was considered important to explore. The modelling could be used to identify which medicines are more likely to be affected by pregnancy and, therefore, would be a priority to obtain clinical data in pregnant women. Eventually, there may be situations where the confidence in the PBPK model is such that it can be used to support extrapolation of efficacy and safety data from healthy volunteers to pregnant women without any clinical data. Ultimately, the hope is that models could be used by healthcare professionals in the clinic to better inform dosing of these patients.

Dosing based solely on exposure, whether measured or predicted, is considered an extrapolation in EU regulatory terms, and a framework has recently been published for children. This framework could be usefully applied to pregnancy (8). A comprehensive PBPK pregnancy model framework could bridge the gaps in data to support a prospective investigation of the exposure, which should then be considered in terms of the exposure–response relationship. This is important for predicting the necessary dose changes to maintain maternal health (9). The first step in the extrapolation is to understand the exposure in pregnancy, and this could be informed by the PBPK models. Such a use would be considered a high-impact application and would require a robust model evaluation.

## Foetal Physiologically Based Pharmacokinetics Models

The evaluation of the risk of foetal exposure to drugs, and its toxicity, during pregnancy is crucial in the benefit–risk assessment of medicines for treating either pre-existing or gestational-related maternal medical conditions. Moreover, in some cases, medical treatment may be needed to prevent vertical disease transmission from the mother to the foetus (e.g., HIV infection). PBPK modelling could provide an understanding of the drug transplacental passage and may be helpful to predict expected foetal medicine levels during pregnancy. The umbilical cord/maternal plasma drug concentration ratio may allow some understanding of the transplacental transfer, although it may not always provide a good prediction of the foetal drug exposure, and the majority of data are in late-stage pregnancy/delivery (10).

## Lactation Physiologically Based Pharmacokinetics Models

Modelling approaches may be useful to estimate mother and infant drug exposure during lactation. PBPK is a potential valuable tool that has been used to predict milk to plasma ratio of potentially harmful medicines and environmental toxins (11). Milk composition varies considerably during and in between breastfeeding sessions, which affects the milk to plasma ratio, leading to variable concentrations of the medicines excreted in milk (12). PBPK may be utilised to estimate the medicine's plasma to milk partition coefficient and calculate the total concentration in milk during breastfeeding, which could be used to calculate the total daily intake by infant. Moreover, PBPK may potentially predict infant exposure during breastfeeding after accounting for the maturation in the drug absorption, distribution and elimination systems (13). The deposition of drug in various infant tissues such as the kidney, liver, and bone marrow may be also simulated.

## Evaluation of the Pregnancy Physiologically Based Pharmacokinetics Model

In a previous publication, we introduced a project to investigate PBPK models to inform drug use in pregnancy (9); this publication includes more detail on what should be considered in terms of model evaluation.

The recommendations in terms of considering whether these models are fit for purpose are outlined in the EMA guideline (6), and key aspects are discussed below. Other frameworks exist for the evaluation of models, e.g., the risk-based credibility assessment framework proposed by Kuemmel et al. (14).

Literature data are available on a number of model drugs that we consider to have rich data sets in pregnancy. For these compounds, drug models are needed; these may be sourced from model repositories; or when models are not available, these models can be built from scratch. In both cases, it is important to determine that the input parameters, i.e., drug physicochemical, PK and PD characteristics, are robust and adequately determined. In the drug development environment in pharmaceutical companies, all input parameters will usually be measured *de novo*; however, when building models retrospectively, it is necessary to source data from the literature; and in some cases, parameters are optimised during model development.

**Figure 1:**
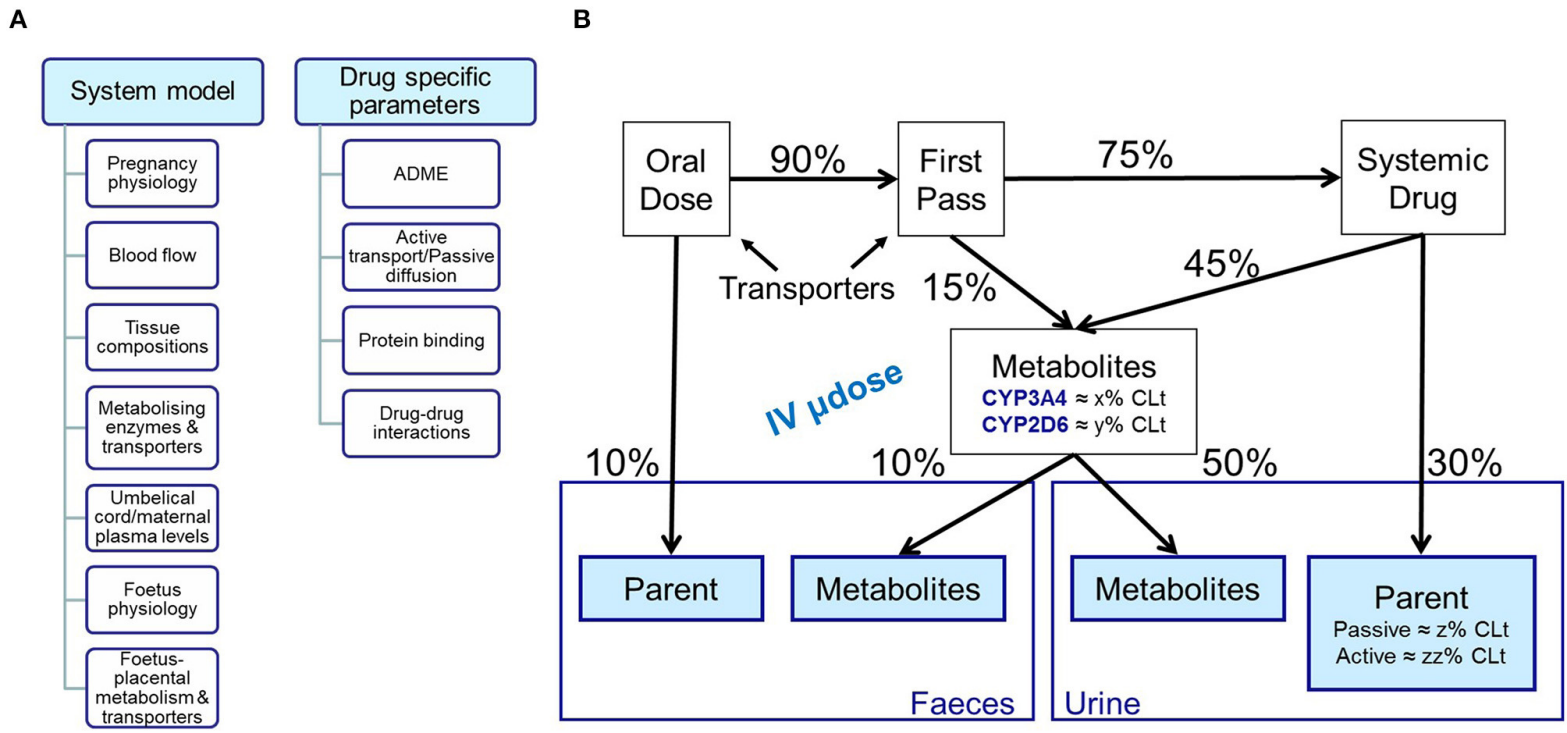
Schematic representation of **(A)** inputs needed for building both system and drug models; **(B)** Mass balance diagram following oral and intravenous dosing.

The important parameters for constructing an appropriate PBPK model depend on the model purpose. In the context of use in pregnancy, an extensive understanding of the absorption and elimination processes is essential, and parameters describing this should be reliable. A drug disposition diagram (15, 16) is recommended for the drugs of interest (Figure 1). The quantitative contribution of all enzymes and transporters involved in the absorption and elimination should be adequately captured in the model, and any uncertainties should be explored with a sensitivity analysis. The quantitation of all pathways to the elimination can be difficult to determine. This may be more challenging for compounds that are substrates of multiple CYPs enzymes, e.g., metronidazole (17), where the specific contribution of each CYP needs to be known, or for substrates of CYP2D6, e.g., metoprolol, as polymorphism may affect the systemic exposure (18). Moreover, the PK of medicines undergoing renal elimination, e.g., amoxicillin, may be altered during pregnancy due to changes in transporters activity/expression; and information about transporters involved in clearance pathways and/or transplacental passage is still limited at the moment (19). The more complex the absorption and elimination processes are for the drug of interest, the more uncertainty this can introduce.

The predictive capability of the drug model should also be determined. The reliability is assessed on the basis of how well important characteristics of the drug model have been tested against *in vivo* PK data. The moiety of interest for predictions should be considered; e.g., parent drug and/or metabolite and the predictive performance of the drug model needs to be demonstrated in healthy volunteers following a range of doses and following single and multiple dosing. Any mis-specification in healthy volunteers will need to be considered when it comes to considering the results in pregnancy.

## Qualification of the Pregnancy Physiologically Based Pharmacokinetics Model

Extensive understanding of physiological changes occurring both during and after pregnancy that may affect drug absorption and disposition (system model) are critical to build a robust PBPK model.

Model qualification is used to determine if these physiology changes have been adequately captured by the system model. In the context of regulatory application, the confidence in the model predictions should be supported by the model qualification, which should be related to the intended purpose and the regulatory impact of the modelling (6, 20). For example, extensive qualification is requested for high-impact models, e.g., when the PBPK is aimed to replace clinical studies or to investigate complex drug–drug interactions (6). A number of compounds with similar ADME characteristics to that of the investigational drug should be used to qualify the model. The confidence in the model depends on both the results of drug model evaluation and the qualification level for the use of the PBPK for the intended purpose (5). For pregnancy PBPK models, clinical PK data in pregnant women collected in all gestational trimesters should be used for the model qualification and validation. However, this might be hampered in some cases due to limited available data in this population, in all trimesters.

For maternal pregnancy models, changes in distribution and elimination are important to capture in the models and to determine the compounds in a qualification data set for a given drug. For example, information about renal changes occurring during all gestational trimesters is crucial for developing a PBPK model aimed to investigate the maternal systemic exposure of medicines undergoing renal elimination. The qualification set should then include drugs renally cleared by the same mechanism. For drugs where a specific enzyme or transporter plays a major role in the absorption, distribution and elimination, the qualification set should consist of a set of drugs for which the same enzyme or transporter plays a significant role. In some cases where multiple enzymes or transporters are involved, then a larger qualification data set may be required including drugs with known maternal exposure, which are substrates for at least one of the enzymes or transporters.

In order to understand foetal exposure of drugs, qualification will be required to show the model's ability to predict foetal concentrations or concentrations entering the foetus. In this situation, qualification will need to focus on the model's ability to predict foetal or cord concentrations.

The approach taken could be a comprehensive model with qualification of maternal and foetal concentrations; alternatively, if the maternal model has already been qualified or concentrations are based on measured values, it is suggested that an abbreviated approach could be taken where the qualification is based on prediction of foetal concentrations when the maternal concentrations are known and foetal concentrations are considered in the terms of a ratio to maternal concentrations. In this case, the qualification set of drugs should include drugs with similar properties to the drug of interest.

In order to understand exposure in milk, qualification using drug concentration PK profile in human milk is normally required to show the model's ability to predict milk concentrations. Comparison between foremilk and hindmilk drug concentrations is recommended to account for any time-dependent changes.

The approach taken could be a comprehensive model with qualification of maternal and foetal concentrations; alternatively, if the maternal model has already been qualified or concentrations are based on measured values, again, an abbreviated approach could be taken where the qualification is based on prediction of milk concentrations when the maternal concentrations are known, in the terms of a ratio. In this case, the qualification set of drugs should include drugs with similar properties to the drug of interest.

## Conclusion

PBPK modelling could be a valuable tool to support the investigation of the expected medicine levels in pregnant women and exposure to the foetus and the infant on breastfeeding and to support the benefit–risk evaluation for drugs to be used in pregnancy.

As a number of gestational age-related physiological changes are expected to alter the PK of medicines during pregnancy, and there are uncertainties on some parameters, well-qualified models are needed to improve assurance in the model prediction before this approach can be used to inform with confidence high-impact decisions as part of regulatory submissions.

## Data Availability Statement

The original contributions generated for the study are included in the article/supplementary material, further inquiries can be directed to the corresponding author/s.

## Author Contributions

All authors listed have made a substantial, direct and intellectual contribution to the work, and approved it for publication.

## Conflict of Interest

The authors are employees of the Medicines and Healthcare products Regulatory Agency, UK.
